# A narrative review on inflammaging and late-onset hypogonadism

**DOI:** 10.3389/fendo.2024.1291389

**Published:** 2024-01-17

**Authors:** Dong Xing, Yihan Jin, Baofang Jin

**Affiliations:** ^1^ Medical College of Southeast University, Nanjing, Jiangsu, China; ^2^ Reproductive Medicine Center, Zhongda Hospital, Southeast University, Nanjing, Jiangsu, China; ^3^ Andrology Department of Integrative Medicine, Zhongda Hospital, Southeast University, Nanjing, Jiangsu, China

**Keywords:** Leydig cell, late-onset hypogonadism (LOH), inflammaging, mitochondrial, senescence-associated secretory phenotype (SASP)

## Abstract

The increasing life expectancy observed in recent years has resulted in a higher prevalence of late-onset hypogonadism (LOH) in older men. LOH is characterized by the decline in testosterone levels and can have significant impacts on physical and mental health. While the underlying causes of LOH are not fully understood, there is a growing interest in exploring the role of inflammaging in its development. Inflammaging is a concept that describes the chronic, low-grade, systemic inflammation that occurs as a result of aging. This inflammatory state has been implicated in the development of various age-related diseases. Several cellular and molecular mechanisms have been identified as contributors to inflammaging, including immune senescence, cellular senescence, autophagy defects, and mitochondrial dysfunction. Despite the extensive research on inflammaging, its relationship with LOH has not yet been thoroughly reviewed in the literature. To address this gap, we aim to review the latest findings related to inflammaging and its impact on the development of LOH. Additionally, we will explore interventions that target inflammaging as potential treatments for LOH.

## 1 Introduction

Aging is a complex biological process characterized by physiological and biochemical changes in the human body. In the male context, a significant outcome of aging is the gradual reduction in the production of testosterone, a pivotal hormone with diverse physiological functions (1). The primary site of testosterone production is the Leydig cells situated in the interstitial tissue of the testes. However, these cells undergo structural and functional alterations with age, leading to decreased testosterone output (2). This reduction in testosterone levels corresponds with a condition termed Late-Onset Hypogonadism (LOH), which is marked by a range of symptoms including diminished libido, fatigue, sarcopenia, and mood fluctuations (3).

Studies indicate that luteinizing hormone (LH) levels remain relatively stable in older males, while testosterone secretion substantially declines (4). Comparative analysis of Leydig cell populations in young and older men reveals a significant reduction in Leydig cell numbers in the elderly (5). Moreover, aging Leydig cells experience disrupted redox balance, leading to intracellular reactive oxygen species (ROS) accumulation and subsequent oxidative stress. This oxidative stress disrupts signaling pathways vital for testosterone synthesis, resulting in reduced expression of key testosterone production molecules and impeding Leydig cell function (6).

Concomitantly, the aging process leads to a chronic low-grade inflammatory state termed inflammaging (7). C-reactive protein, interleukin-6, tumor necrosis factor-α, interleukin-1β, and other related markers are routinely utilized as straightforward serum biomarkers for assessing inflammaging (8). Circulating miRNA sequencing and quantification of circulating cell-free mitochondrial DNA copy numbers are also approaches for assessing systemic inflammation (9, 10). Unlike acute inflammation, inflammaging persists over time and is closely linked to the onset of several chronic and age-related ailments (8). Numerous studies consistently demonstrate elevated levels of inflammatory factors like TNF-α, IL-1β, and IL-6 in older males’ circulation (11–13). The activation of inflammatory signaling pathways, predominantly orchestrated by NF-KB and P38MAPK, assumes a central role in cellular aging. These pathways’ activation prompts the release of inflammatory mediators and proteases, culminating in the formation of the Senescence-Associated Secretory Phenotype (SASP) (14). Due to the lack of a protective blood-testis barrier, these inflammatory cytokines can easily enter the testicular interstitium through circulation, adversely affecting Leydig cell function (15). Additionally, the aging process can prompt the shift of M2-type testicular macrophages to pro-inflammatory M1-type testicular macrophages, releasing inflammatory cytokines and exacerbating testicular interstitial inflammation (16).

This article offers a comprehensive overview of recent research on inflammaging, immune senescence, cellular senescence, mitochondrial dysfunction, and the pathophysiological progression of LOH. Furthermore, we delve into the potential of targeting inflammaging as a promising strategy for future therapeutic interventions to slow down or halt the progression of age-related LOH.

## 2 Regulation of testosterone synthesis in Leydig cells

Testosterone synthesis within Leydig cells is primarily orchestrated by the hypothalamic-pituitary-gonadal (HPG) axis (17). The hypothalamus releases gonadotropin-releasing hormone (GnRH) in rhythmic pulses (18). GnRH interacts with pituitary gonadotroph receptors, stimulating LH secretion from the pituitary gland (19). LH binds to Leydig cell receptors, activating adenylyl cyclase. This triggers ATP to cyclic adenosine monophosphate (cAMP) conversion, activating Protein Kinase A (PKA) downstream (20). Cholesterol then enters Leydig cells, converting to free cholesterol. Steroidogenic acute regulatory protein (StAR) transports free cholesterol to mitochondria. P450scc enzyme in mitochondria cleaves free cholesterol to form pregnenolone (21, 22). Pregnenolone moves to the endoplasmic reticulum, becoming progesterone and dehydroepiandrosterone via enzymes like 3β-hydroxysteroid dehydrogenase (3β-HSD) and cytochrome P450 17A1 (CYP17A1) (23). Both progesterone and dehydroepiandrosterone are subsequently transformed into androstenedione. Ultimately, testosterone synthesis is realized through the catalytic action of 17β-hydroxysteroid dehydrogenase (17β-HSD) (24). During the aging process, certain changes in the body may affect testosterone synthesis by influencing the function of the HPG axis.

## 3 Immuno-senescence

Immuno-senescence, the gradual decline in immune system function with age, is a pivotal factor in the aging process (25). It involves reduced immune cell abundance, compromised efficiency, altered response patterns, heightened inflammation, and reduced tolerance (26). The aging process affecting peripheral blood immune cells can induce systemic inflammation, potentially disrupting the stable functioning of the hypothalamic-pituitary-gonadal (HPG) axis and resulting in impaired regulation of testosterone synthesis. Furthermore, the aging of testicular macrophages may contribute to localized inflammation, directly impeding the normal functionality of Leydig cells.

### 3.1 Changes in peripheral blood immune cells

Aging immune cells contribute to systemic inflammation and age-related disorders (27). Huang et al.’s single-cell sequencing study on human immune profiles across aging and gender revealed significant shifts in peripheral blood immune cells, contributing to systemic inflammation (28).

#### 3.1.1 T cell alterations in aging males

T cells are the most significantly altered immune cell type in the peripheral blood of elderly males, characterized by a marked decrease in quantity, significant features of cellular aging, and upregulation of inflammatory gene expression. Firstly, single-cell sequencing studies suggest a significant reduction in the numbers of CD8+ T cells and CD4+ naïve T cells in the peripheral blood of elderly males (28). Martinez-Zamudio et al. noted senescent features in CD8+ T cells, including elevated intracellular Senescence-associated beta-galactosidase (SA-βGal) activity, telomere dysfunction, and impaired mitochondrial transcription factors in older males (29). In addition, elderly males exhibit elevated expression of specific genes like DUSP2, CXCR4, DDIT4, NF-KBIA, and JUNB in CD8+ T cells and CD4+ T cells, along with the activation of MAPK signaling pathways (28). These changes impair T cell function, causing reduced proliferation, increased lysosomal content, and excess pro-inflammatory cytokines (30, 31).

#### 3.1.2 Natural killer cell dynamics in aging males

Natural Killer (NK) cells, crucial components in innate immunity, express CD56 and CD16 (32). Based on CD56 expression, they can be classified into CD56^bright^CD16+ and CD56^dim^CD16+ subpopulations. CD56^bright^CD16+ NK cells possess cytokine secretion capabilities and regulate immunity, while CD56^dim^CD16+ NK cells exhibit higher cytotoxicity and mainly engage in innate immunity (33). Aging affects these NK cell subsets in men’s peripheral blood, reducing CD56^bright^CD16+ and increasing CD56dimCD16+ NK cell absolute numbers and proportions (34). Moreover, a subset of CD56^dim^CD16+ NK cells has been observed to express elevated levels of CD57, a marker associated with highly differentiated cells (35). Some CD56dimCD16+CD57+ cells exhibit heightened expression of senescence-associated genes like ZFP36 and DUSP2 (28), elevating cytotoxicity and cytokine secretion (IL-6, TNF-α) (36–38).

#### 3.1.3 Monocyte changes in aging males

The influence of aging on peripheral blood monocyte counts remains debated. Seidler S et al.’s study suggests minimal impact (39). However, recent sequencing studies reveal increased monocyte levels in aging individuals (28). However, multiple studies have consistently concluded that aging leads to an increase in the number of CD16+ monocytes, indicating phenotypic alterations in monocytes during the aging process (28, 39–41). Unlike classical CD14+ monocytes, CD16+ monocytes have an enhanced inflammatory potential that promotes the development of the SASP (42, 43). Simultaneously, the NF-KB signaling pathway, IL-1 signaling pathway, and inflammatory response signaling pathway in monocytes were markedly activated, with a concomitant significant upregulation in the expression of pro-inflammatory genes TNF, JUNB, and DDIT4 in aging men (28).

#### 3.1.4 Dendritic cell dynamics in aging males

Dendritic cells play a pivotal role in the immune response as they serve as antigen-presenting cells to T cells. They encompass plasmacytoid dendritic cells (pDCs) and myeloid dendritic cells (mDCs) (44). Most scientific investigations have consistently concluded that the quantity of dendritic cells in the peripheral blood of healthy older adults does not exhibit noteworthy changes when compared to their younger counterparts (28, 45, 46). Nevertheless, shifts in pDCs and mDCs proportions during senescence remain debated (47–49). Regardless of mDCs abundance changes, they exhibit pronounced pro-inflammatory characteristics compared to pDCs. Senescent mDCs exhibit reduced phosphoinositide 3-kinase (PI3K) activity, amplifying NF-KB activation and subsequent pro-inflammatory cytokine generation (45). Senescent mDCs also show elevated CD68 expression, indicating sustained activation, leading to heightened pro-inflammatory cytokine production even without external stimuli (50).

### 3.2 Changes of testicular macrophages

#### 3.2.1 Function of testicular macrophages

Testicular macrophages are pivotal in maintaining testicular immune privilege, supporting spermatogenesis, and modulating testosterone synthesis (51–53). They are categorized into interstitial and peritubular macrophages based on phenotypic traits (54). These cells originate from embryonic and bone marrow progenitors, adopting an anti-inflammatory state with TGF-β and IL-10 secretion to support testosterone synthesis and spermatogenesis (52, 55). Furthermore, these macrophages possess the capability to secrete 25-hydroxycholesterol, facilitating the process of testosterone synthesis in Leydig cells (56).

#### 3.2.2 Influence of aging on testicular macrophages

In early investigations, transmission electron microscopy was employed to examine age-related alterations in testicular macrophages, revealing abnormalities in cellular structures such as mitochondria and the Golgi apparatus (57). However, the mechanisms underlying these changes in aging macrophages remained unclear due to methodological limitations. With the advent and progression of sequencing technologies, we have attained a more comprehensive understanding of the genetic, phenotypic, and quantitative changes in aging macrophages. Aging increases pro-inflammatory macrophages. Nie et al. identified elevated hyperactivated (M1-type) macrophages and heightened cytokine secretion in elderly human testes (58). Mouse research supported these findings, revealing three macrophage subpopulations. Subpopulation 3 (senescence-specific) increased significantly in aged mice, showing a hyperactivated state and inflammation-related gene expression. Aging enriched genes related to subpopulations 1 and 2, linked to type I interferon secretion and Toll-like receptor 4 signaling (16).

### 3.3 Effect of immuno-senescence on HPG axis

**Figure 1:**
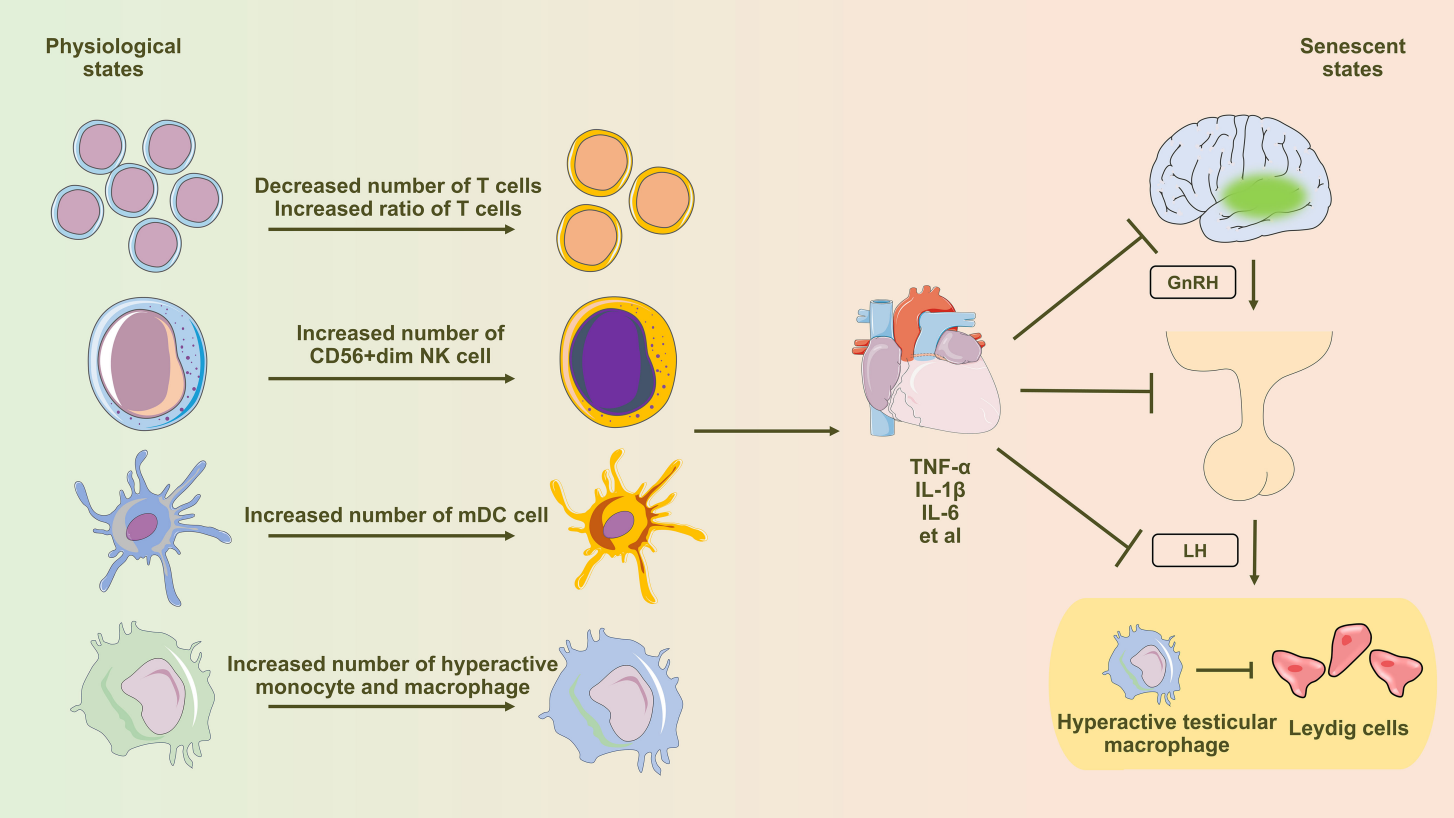
Diagram illustrating the influence of immune senescence-induced inflammaging on testosterone synthesis in Leydig cells. Aging profoundly affects peripheral blood immune cells, inducing notable alterations in T cells, NK cells, dendritic cells, monocytes, and macrophages. This shift leads to an elevated presence of pro-inflammatory immune cells and an excessive production of pro-inflammatory cytokines, subsequently influencing the regular functionality of the hypothalamic-pituitary-gonadal (HPG) axis via the circulatory system. Testicular macrophages, under the influence of senescence, transition towards the M1 subtype (hyperactivated), characterized by heightened secretion of abundant pro-inflammatory factors. These factors are then released through paracrine signaling, directly impacting the functionality of Leydig cells.

Alterations in peripheral blood immune cells during immunosenescence significantly contribute to heightened proinflammatory status in men. This senescent inflammatory state profoundly impacts the HPG axis function (59). The aging process of the hypothalamus is intricately linked to the activation of the NF-KB signaling pathway. The activation of the NF-KB signaling pathway exerts an impact on the functionality of Gonadotropin-Releasing Hormone (GnRH) neurons, impeding GnRH gene transcription and culminating in an anomalous release of GnRH within the hypothalamus (60). Studies have identified an augmented presence of pro-inflammatory cytokines, particularly IL-6, in the pituitary tissue of aging mice. This inflammatory condition markedly hinders the tissue repair mechanisms of pituitary stem cells (61). Aging notably expands pro-inflammatory testicular macrophages, increasing pro-inflammatory cytokine secretion and gene expression. This macrophage shift significantly influences the testicular inflammatory microenvironment during aging (62). Systemic and localized inflammation may exacerbate Leydig cellular aging, leading to mitochondrial dysfunction and inhibition of autophagy (**Figure 1**).

## 4 Cellular senescence

Cellular senescence is characterized by the cessation of cell proliferation and functionality under specific circumstances, accompanied by distinct alterations in morphology, physiology, and molecular attributes (63). This intricate biological phenomenon is intricately linked to the typical progression of organisms, the aging trajectory, and the onset of various diseases.

### 4.1 Basic change of senescent Leydig cells

The pioneering work of Hayflick and Moorhead unveiled senescent cells’ inability to undergo customary cell division as essential for cell cycles (64). Subsequent investigations have encompassed telomere length and telomerase function as principal determinants contributing to cell cycle arrest (65, 66). Morphological and structural changes often characterize senescent cells, manifesting as an enlarged, flattened morphology and irregular organelles (67). SA-βGal serves as a widely employed biomarker, offering insights into the extent of cellular senescence to some extent, although not serving as an obligatory indicator (68). Simultaneously, the expression of specific cell cycle inhibitory proteins, notably p16 and p21, experiences significant upregulation in senescent cells (69). Jin et al. employed transmission electron microscopy to detect find fewer, disorganized mitochondria in senescent Leydig cells (70). Jeong et al. observed pronounced elevation in SA-βgal levels, along with heightened expressions of p16, p19, and p21, in aging Leydig cells (71).

### 4.2 SASP in senescent Leydig cells

SASP is crucial in cellular aging, where senescent cells release various molecules affecting the microenvironment and nearby cells (72). Persistent SASP can trigger inflammation and age-related disorders (73–75).

#### 4.2.1 Activation of NF-KB pathways

The NF-KB signaling pathway is pivotal in immune responses, inflammation, proliferation, and apoptosis (76). Activation pathways typically involve cell surface receptors, viral infections, bacterial components, and cytokines (77). NF-KB translocates from cytoplasm to nucleus, binding SASP-related gene promoters, inducing transcription (78). Furthermore, NF-KB can indirectly enhance the expression of the transcription factor C/EBPβ, subsequently facilitating direct regulation of SASP gene expression by C/EBPβ (79, 80). Within Leydig cells, Tao Shang and colleagues demonstrated significant activation of the NF-KB signaling pathway (81). Upon exposure to inflammatory induction, mouse Leydig progenitor cells and the TM3 cell line exhibited a substantial increase in the levels of pro-inflammatory cytokines in the cell culture supernatants.

#### 4.2.2 Activation of MAPK pathways

The MAPK signaling pathway, including ERK1/2, JNK, and P38 MAPK, is pivotal in cell processes like proliferation, differentiation, apoptosis, and inflammation (82, 83). Adam Freund et al. suggested P38MAPK’s autonomous role in SASP, enhancing NF-KB’s transcriptional activity for SASP activation (84). Simultaneously, P38MAPK exerts an indirect influence by potentiating the activation of transcription factors such as ATF2, AP-1, and CREB1 (85, 86). Consequently, these activated transcription factors interplay in the regulation of SASP. P38MAPK activation was found in aging Leydig cells, while ERK1/2 or JNK activation wasn’t prominent (87). This suppresses key testosterone synthesis molecules expression in Leydig cells, ultimately reducing serum testosterone levels.

The components of SASP secreted by senescent Leydig cells can exert paracrine effects on neighboring cells (88). It is noteworthy that the C/EBPβ, ATF2, AP-1, and CREB1 molecules belong to the family of basic leucine zipper transcription factors (89). Indeed, these molecules are intricately involved in the regulation of SASP (90). Importantly, within Leydig cells, they assume a pivotal role in orchestrating testosterone synthesis pathways (91, 92).

## 5 Mitochondrial dysfunction

Leydig cellular senescence is closely linked with mitochondrial dysfunction. Aging induces oxidative stress in cells, thereby influencing the expression levels of crucial molecules involved in testosterone synthesis on the mitochondria. This, in turn, initiates processes such as apoptosis and necroptosis, culminating in mitochondrial dysfunction.

### 5.1 Role of mitochondria in testosterone synthesis

Mitochondria, vital intracellular organelles, play a crucial role in testosterone synthesis within Leydig cells. Firstly, they are central to generating ATP, the essential energy substrate required for testosterone synthesis (93). Secondly, mitochondria are integral to the intricate signaling cascade governing testosterone synthesis, as key molecules like StAR and CYP11A1 localize within these organelles (94–96). Additionally, mitochondria regulate oxidative stress and apoptosis in Leydig cells, highlighting their multifaceted involvement (97, 98).

### 5.2 Mitochondrial dysfunction in senescent Leydig cells

Mitochondrial dysfunction significantly contributes to impaired testosterone synthesis in senescent Leydig cells (99). The expression of StAR on the outer mitochondrial membrane of senescent Leydig cells shows a notable reduction (100), leading to structural perturbations in Leydig cell mitochondria and subsequent impairment of mitochondrial oxidative respiratory chain function (101). Dysfunctional mitochondria in Leydig cells can lead to reduced ATP synthesis and an accumulation of intracellular ROS (102). Excess ROS triggers the activation of inflammatory vesicles, initiating cellular pyroptosis (103). This process involves the NLRP3 inflammasome, where NF-KB activation, in response to inflammatory stimuli, amplifies the transcription of inflammasome-related genes (104). NLRP3 activation recruits the adaptor molecule ASC, initiating caspase-1 activation. Caspase-1 cleaves pro-IL-1β into IL-1β and pro-IL-18 into IL-18, resulting in their secretion and subsequent apoptosis (105). In response to inflammatory stimuli, Leydig cells exhibit increased intracellular NLRP3 and ASC expression, caspase-1 cleavage, conversion of IL-1β and IL-18, apoptosis activation, and reduced expression of testosterone-synthesizing molecules (106). Collectively, these changes culminated in the inhibition of testosterone synthesis.

## 6 Autophagy deficiency

### 6.1 Role of autophagy in Leydig cells

Autophagy, an intrinsic catabolic process, maintains intracellular stability by degrading impaired cellular components (107). Eukaryotic cells primarily classify autophagy into three major types: macroautophagy, microautophagy, and chaperone-mediated autophagy (108). Given its specificity toward organelles and cellular contents, autophagy further subdivides into types such as mitophagy, ER-phagy, and lipophagy (109–111). In Leydig cells, autophagy regulates oxidative stress, cholesterol uptake, apoptosis, and pyroptosis, influencing testosterone synthesis capacity (106, 112–114).

### 6.2 Autophagy deficiency and its consequences in Leydig cells

**Figure 2:**
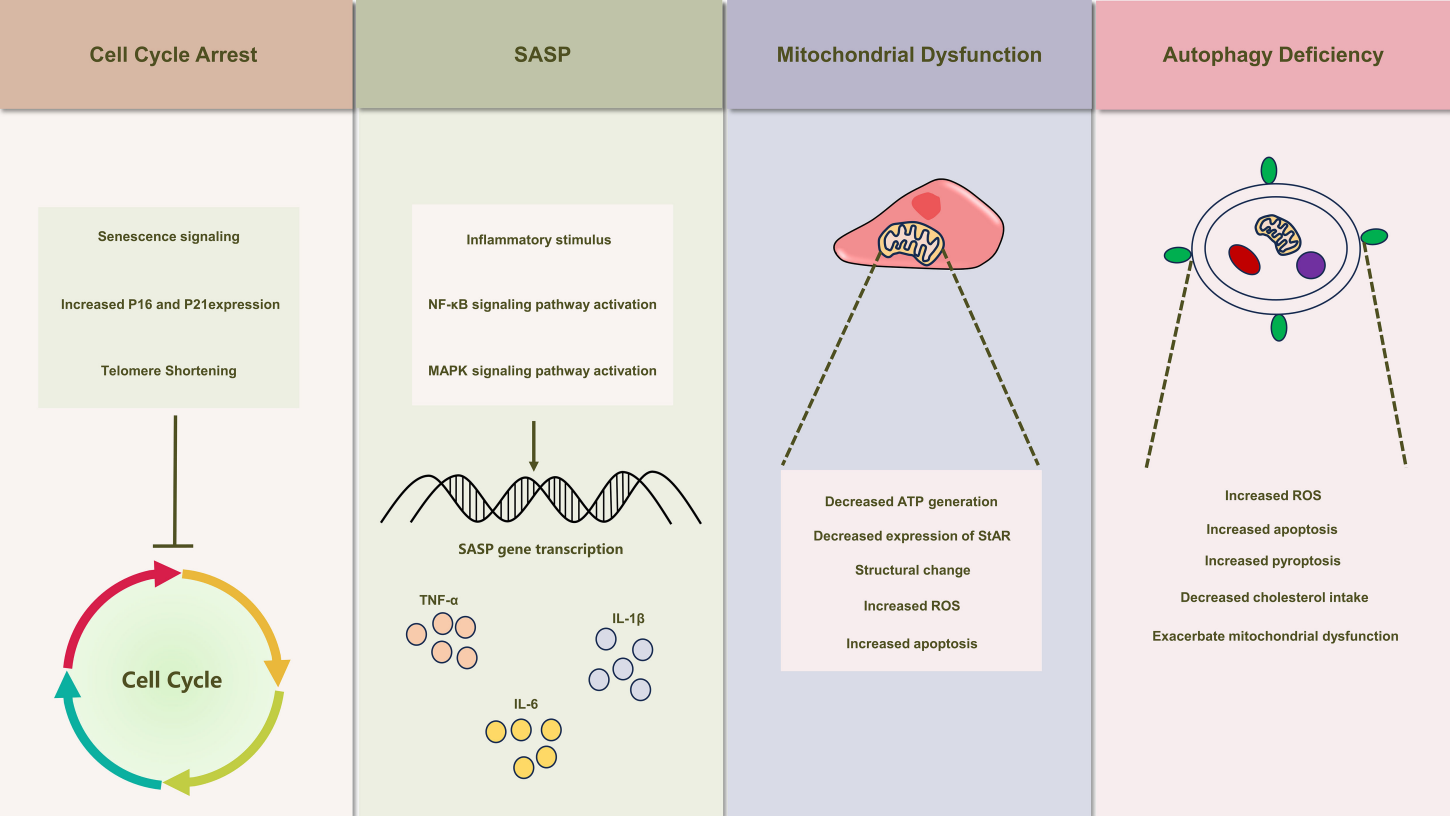
Illustrates the schematic depiction of senescence-induced alterations in Leydig cells. Leydig cell senescence manifests a spectrum of modifications, encompassing cell cycle arrest, the emergence of senescence-associated secretory phenotypes (SASP), mitochondrial dysfunction, and autophagy deficiency. These alterations also play a crucial role in the development of testicular inflammaging, leading to substantial inhibition of Leydig cell function.

Mitochondria exhibit dynamic behavior within the cell, continuously undergoing fusion and division processes (115). The occurrence of mitophagy is intricately linked to the processes of mitochondrial fusion and division. Mitochondrial fission stimulates the expression of pre-mitochondrial fission proteins, thereby triggering a corresponding alteration in the extent of mitophagy (116). Recent research on Leydig cells highlighted elevated N6-methyladenosine (m6A) levels in primary Leydig cells from senescent mice. This m6A increase impedes intracellular autophagy, influencing testosterone synthesis functionality (117). Reduced autophagy induces redox imbalance in Leydig cells, accumulating intracellular ROS (114). Dysregulated ROS levels disturb mitochondrial fusion equilibrium, impacting autophagy. Yi et al. attributed cadmium-induced apoptosis in Leydig cells to mitochondrial fragmentation, causing impaired function and elevated superoxide and ROS levels, inhibiting mitochondrial autophagy (118). Diminished autophagy in Leydig cells also links to pyroptosis initiation. Inflammatory stimuli reduce autophagy, activating cellular inflammasome and initiating pyroptosis (106) (**Figure 2**).

## 7 Possible therapeutic strategies

The established clinical intervention for LOH is testosterone replacement therapy (TRT). Nevertheless, TRT is accompanied by unavoidable side effects (119). We present various alternative interventions, different from TRT, capable of ameliorating LOH symptoms by mitigating inflammaging (**Table 1**).

**Table 1:** Interventions targets inflammaging of LOH.

	Mechanisms	Interventions	References
Physical exercise	alleviate systemic inflammation, enhance cellular antioxidant stress capacity, and elevate testosterone levels	Aerobic exercise and weight training	(123–125)
Anti-inflammation Antioxidant Interventions	Diminishes cellular oxidative stress alleviates inflammation	Adrenomedullin SS-31	(126–129, 133–135)
Cell Transplantation	Facilitates testicular structural restoration ameliorates testicular senescence	Leydig cell transplantation Leydig stem cell transplantation stem cell transplantation	(140–142)
Traditional Chinese Medicine and Its Extract	mimics sex hormones Enhance the expression of molecules associated with testosterone synthesis Diminishes cellular oxidative stress alleviates inflammation	Yangjing Capsules Icariin Polygonatum sibiricum polysaccharides Ginseng saponin Ashwagandha	(143–145, 152)

### 7.1 Physical exercise

Physical exercise emerges as a natural and comprehensive approach to health management, demonstrating a visible impact on clinical symptoms associated with various diseases (120). In the context of LOH, engagement in suitable physical exercise holds the potential to alleviate systemic inflammation, enhance cellular antioxidant stress capacity, and elevate testosterone levels in patients. In a randomized controlled trial involving individuals aged 45-75, participation in interval aerobic exercise at an intensity reaching 90% of the maximum heart rate three times a week yielded a substantial reduction in the circulating levels of C-reactive protein and TNF-α in the study participants (121). Several clinical trials have substantiated that older individuals participating in regular physical exercise manifest heightened levels of antioxidant capacity markers in their blood, including glutathione peroxidase, total nitrite/nitrate, and total oxyradical scavenging capacity, in comparison to their sedentary counterparts (122). Moreover, moderate-intensity aerobic exercise has demonstrated a noteworthy capacity to substantially elevate testosterone levels in elderly men (123).

### 7.2 Anti-inflammation and antioxidant interventions

Adrenomedullin (ADM), a peptide hormone synthesized primarily in the adrenal medulla, has a broader presence, including the testes (124, 125). ADM exerts potent protective effect in inflammatory contexts (126). Rat primary Leydig cells synthesize ADM and express its receptors, suggesting its autocrine and paracrine roles in safeguarding testosterone production (127). ADM potentially protects Leydig cells during inflammation by curbing ROS production, stabilizing mitochondria, inhibiting MAPK and NF-KB signaling, and enhancing autophagy, which counters apoptosis and pyroptosis (128, 129). Mitochondria-targeted antioxidants interact directly with mitochondria to counteract oxidative stress and damage (130). SS peptides, specifically SS-31, have been extensively researched for their impact on mitochondrial function (131). SS-31 operates at the cellular level, reducing and neutralizing ROS generation, effectively mitigating oxidative stress within cells and mitochondria (132). It plays a critical role in maintaining mitochondrial membrane integrity, thereby balancing mitochondrial fusion and fission, vital for cellular energy metabolism and function (133). Evidence also suggests that SS-31 exhibits anti-inflammatory properties, modulating inflammatory mediator production and cellular responses to inflammation (134). Its potential spans various inflammatory and age-related conditions, including cardiovascular diseases, kidney ailments, and Alzheimer’s disease (135, 136). Currently, clinical trials for the therapeutic application of ADM and SS-31 in specific diseases have progressed to phase II trials (137). Further investigations are imperative to establish the safety profile of these drugs in clinical settings and ascertain its efficacy for addressing LOH.

### 7.3 Cell transplantation

In recent years, cell transplantation has garnered growing attention as a therapeutic approach for a diverse range of diseases. Cell transplantation methods employed for addressing LOH encompass Leydig cell transplantation, Leydig stem cell transplantation, and stem cell transplantation. Luo et al. conducted a study that introduced an autofluorescence-based technique for the isolation and purification of Leydig cells. Subsequently, these isolated cells were transplanted subcutaneously into denuded mice, resulting in elevated testosterone levels (138). ARORA et al. demonstrated that the concurrent subcutaneous transplantation of Leydig stem cells, supporting cells, and myoid cells facilitated the differentiation and maturation of Leydig cells, leading to the secretion of testosterone (139). In rats, tail vein injection of bone marrow mesenchymal stem cells (MSCs) has been observed to elevate serum testosterone levels. This effect is attributed to the reduction of oxidative stress and senescence phenotype in Leydig cells (140).

### 7.4 Traditional medicine

Several herbal remedies from Traditional Chinese Medicine, aiming to enhance sex hormone levels and male sexual function, show promise in addressing LOH. Our team developed “Yangjing Capsules,” demonstrating positive impacts on Leydig cell testosterone synthesis (141, 142). Complex herbal compositions pose challenges in pinpointing therapeutic elements and understanding clinical efficacy. Recent research focuses on individual herbs and extracts, verifying direct effects on Leydig cell testosterone synthesis. Icariin, from Epimedium, mimics sex hormones, enhances immunity, and reduces inflammation (143, 144). Studies confirm icariin stimulates testosterone synthesis through pathways involving the upregulation of testosterone synthesis-associated molecules, mitochondrial function preservation, and other mechanisms (145, 146). Polygonatum sibiricum polysaccharides, extracted from Polygonatum sibiricum, renowned for kidney and sexual function enhancement, exhibit antioxidative and anti-inflammatory effects (147). These polysaccharides safeguard testosterone synthesis in mice by reducing oxidative stress, preserving mitochondrial function, and inhibiting apoptosis (148). Additionally, a range of herbs has been explored for their potential to support and protect testosterone synthesis (149), warranting comprehensive investigation across various experimental domains. Ashwagandha, commonly referred to as Withania somnifera, is an herb extensively employed in Indian traditional medicine. It holds promise in ameliorating symptoms associated with LOH through potential benefits, encompassing stress reduction, antioxidant properties, anti-inflammatory effects, immune modulation, and facilitation of testosterone synthesis, among other mechanisms (150–152).

## 8 Conclusions

**Figure 3:**
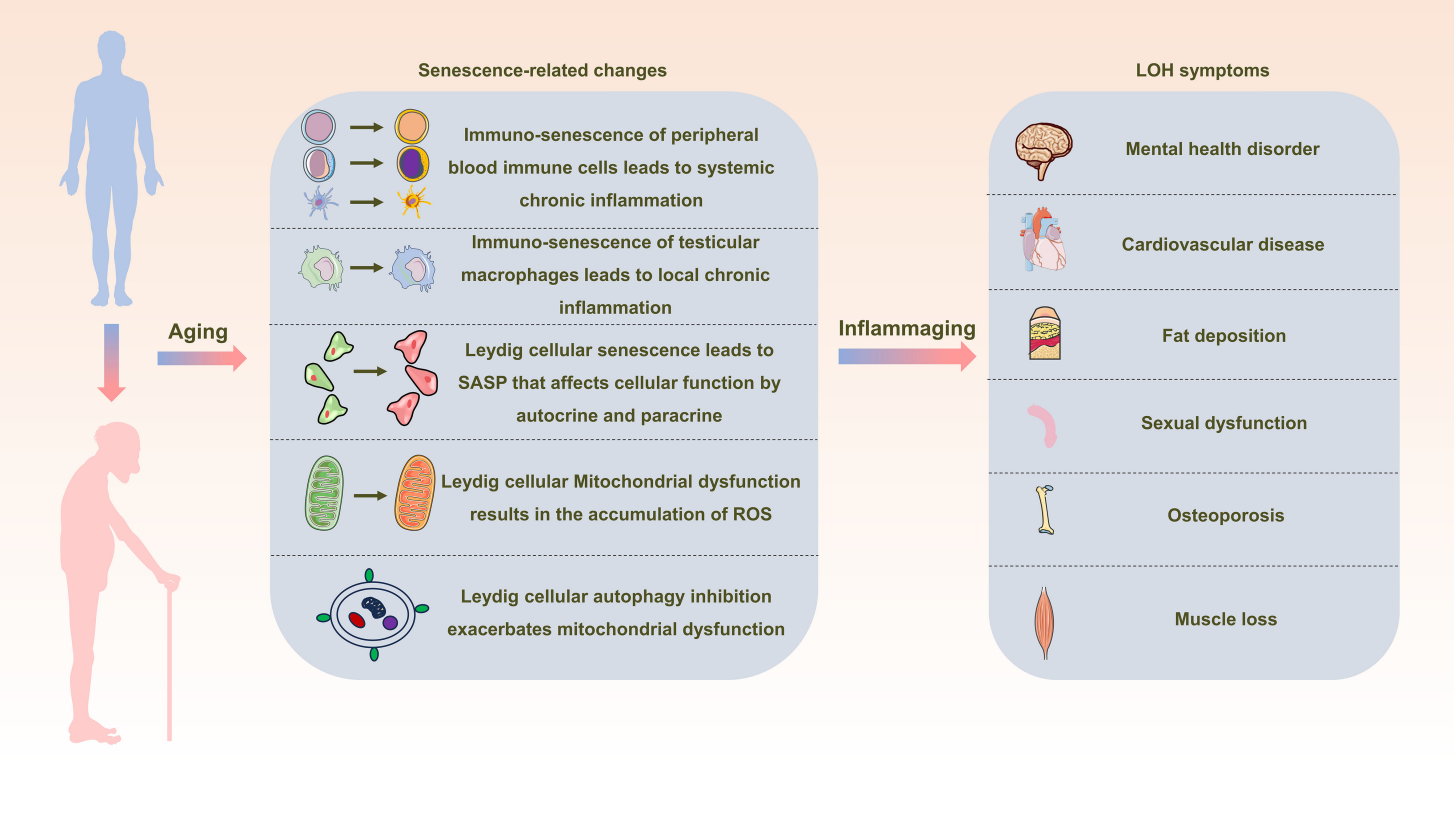
Schematic representation depicting the interrelation between inflammaging and late-onset hypogonadotropic hypogonadism (LOH). As aging progresses, alterations in peripheral blood immune cells, testicular macrophages, and Leydig cells become pronounced, creating a milieu of widespread and localized inflammation. This state, termed inflammaging, disrupts the intricate process of testosterone synthesis within Leydig cells, consequently initiating and advancing the development of LOH in aging males.

In the elderly population, chronic aseptic low-grade inflammation, referred to as inflammaging, contributes to the emergence and progression of diverse age-associated conditions. Both systemic and localized immuno-senescence expose Leydig cells to an inflammatory milieu. Simultaneously, SASP stemming from Leydig cellular senescence, along with factors such as mitochondrial dysfunction and impaired autophagy, amplify the inflammatory response within Leydig cells. These combined factors culminate in the impairment of testosterone synthesis function, sparking the onset of LOH (**Figure 3**). Notably, investigations concerning the influence of treatments targeting inflammation and mitochondrial function on LOH remain limited. This research gap assumes paramount significance in fulfilling the medical necessities and enhancing the well-being of elderly men, warranting extensive further exploration and translational efforts.

## Author contributions

DX: Conceptualization, Writing – original draft, Writing – review & editing. YJ: Funding acquisition, Writing – review & editing. BJ: Funding acquisition, Writing – review & editing.
